# Supplementary material for: Mechanisms and impact of public reporting on physicians and hospitals’ performance: A systematic review (2000–2020)
Source: PLoS One. 2021 Feb 24;16(2):e0247297. doi: 10.1371/journal.pone.0247297 (PMC7904172; doi:10.1371/journal.pone.0247297)
Supplement: S1 Appendix — (DOCX) [file pone.0247297.s002.docx]

**S1 Appendix**

**Medline search strategy**

1. (public adj2 (report* or disclosure* or audit* or information)).mp.

2. (performance adj2 (report* or outcome* or indicator* or measure* or data or information or rating)).mp.

3. (report adj (card or cards)).mp.

4. (consumer adj2 (report* or information)).mp.

5. (quality adj2 (indicator* or information or criteria or criterion or standard* or norm)).mp.

6. benchmarking.mp.

7. provider profiling.mp.

8. (public release and (data or information or performance)).mp.

9. (accreditation adj2 report*).mp.

10. or/1-9

11. (random* adj2 (trial* or study)).mp. or randomized controlled trial.pt.

12. clinical trial.mp. or clinical trial.pt. or multicenter studies.pt. or evaluation studies.pt.

13. ((time adj series) or longitudinal study or longitudinal studies).mp.

14. quasi.mp.

15. ("before and after" or pre test or pretest or posttest or post test).mp.

16. (cohort study or cohort studies).mp.

17. case control.mp.

18. (cross sectional or cross-sectional).mp.

19. or/11-18

20. 10 and 19

21. consumer satisfaction.mp.

22. patient preference*.mp.

23. decision making.mp.

24. (choice adj2 (behavior or behaviour)).mp.

25. patient acceptance of health care.mp.

26. patient participation.mp.

27. patient satisfaction.mp.

28. patient attitude.mp.

29. (utilisation or utilization).mp.

30. (purchasing or funding or buying).mp.

31. governance.mp.

32. physicians practice.mp.

33. (clinical practice or medical practice or healthcare quality).mp.

34. (quality adj2 improv*).mp.

35. ((outcome* adj2 improve*) or (patient* adj2 outcome*)).mp.

36. ((organisation* or organization*) adj2 (change* or process* or development or structur*)).mp.

37. clinical outcome*.mp.

38. (adverse effect* or ((unintended or dysfunctional or negative) adj2 (effect* or consequence* or outcome*))).mp.

39. (health care quality or health care planning).mp.

40. or/21-39

41. exp primary health care/

42. exp hospitals/

43. physicians/

44. health professionals.ab,ti.

45. health personnel/

46. health plans.ab,ti.

47. health plan.ab,ti.

48. insurance.ab,ti.

49. (physician* or gp or gps or doctor or doctors or general practi* or prescriber* or group pract* or institutional pract* or partnership pract* or family pract* or office pract* or private pract* or primary pract* or nurse or nurses).tw.

50. (pharmacist* or pharmacies or pharmacy).tw.

51. hospital*.tw.

52. physiotherapist.mp.

53. midwife.mp.

54. (health care centre* or health care center* or health care system* or healthcare centre* or healthcare center* or healthcare system* or health center* or health centre* or health system* or (health adj2 organisation*) or (health adj2 organization*)).mp.

55. (medical centre* or medical center* or medical system*).mp.

56. dietician.mp.

57. (health care provider* or healthcare provider* or medical provider*).mp.

58. psychologist.mp.

59. psychiatrist*.mp.

60. exp group practice/

61. exp institutional practice/

62. (dentists or dental clinics).mp.

63. exp private practice/

64. exp family practice/

65. exp physicians/

66. exp physicians, family/

67. exp professional practice/

68. exp nurses/

69. exp nurse clinicians/

70. physician's practice patterns/

71. or/41-70

72. 20 and 40 and 71

73. limit 72 to english

**S1 Appendix**

**Medline search strategy**

1. (public adj2 (report* or disclosure* or audit* or information)).mp.

2. (performance adj2 (report* or outcome* or indicator* or measure* or data or information or rating)).mp.

3. (report adj (card or cards)).mp.

4. (consumer adj2 (report* or information)).mp.

5. (quality adj2 (indicator* or information or criteria or criterion or standard* or norm)).mp.

6. benchmarking.mp.

7. provider profiling.mp.

8. (public release and (data or information or performance)).mp.

9. (accreditation adj2 report*).mp.

10. or/1-9

11. (random* adj2 (trial* or study)).mp. or randomized controlled trial.pt.

12. clinical trial.mp. or clinical trial.pt. or multicenter studies.pt. or evaluation studies.pt.

13. ((time adj series) or longitudinal study or longitudinal studies).mp.

14. quasi.mp.

15. ("before and after" or pre test or pretest or posttest or post test).mp.

16. (cohort study or cohort studies).mp.

17. case control.mp.

18. (cross sectional or cross-sectional).mp.

19. or/11-18

20. 10 and 19

21. consumer satisfaction.mp.

22. patient preference*.mp.

23. decision making.mp.

24. (choice adj2 (behavior or behaviour)).mp.

25. patient acceptance of health care.mp.

26. patient participation.mp.

27. patient satisfaction.mp.

28. patient attitude.mp.

29. (utilisation or utilization).mp.

30. (purchasing or funding or buying).mp.

31. governance.mp.

32. physicians practice.mp.

33. (clinical practice or medical practice or healthcare quality).mp.

34. (quality adj2 improv*).mp.

35. ((outcome* adj2 improve*) or (patient* adj2 outcome*)).mp.

36. ((organisation* or organization*) adj2 (change* or process* or development or structur*)).mp.

37. clinical outcome*.mp.

38. (adverse effect* or ((unintended or dysfunctional or negative) adj2 (effect* or consequence* or outcome*))).mp.

39. (health care quality or health care planning).mp.

40. or/21-39

41. exp primary health care/

42. exp hospitals/

43. physicians/

44. health professionals.ab,ti.

45. health personnel/

46. health plans.ab,ti.

47. health plan.ab,ti.

48. insurance.ab,ti.

49. (physician* or gp or gps or doctor or doctors or general practi* or prescriber* or group pract* or institutional pract* or partnership pract* or family pract* or office pract* or private pract* or primary pract* or nurse or nurses).tw.

50. (pharmacist* or pharmacies or pharmacy).tw.

51. hospital*.tw.

52. physiotherapist.mp.

53. midwife.mp.

54. (health care centre* or health care center* or health care system* or healthcare centre* or healthcare center* or healthcare system* or health center* or health centre* or health system* or (health adj2 organisation*) or (health adj2 organization*)).mp.

55. (medical centre* or medical center* or medical system*).mp.

56. dietician.mp.

57. (health care provider* or healthcare provider* or medical provider*).mp.

58. psychologist.mp.

59. psychiatrist*.mp.

60. exp group practice/

61. exp institutional practice/

62. (dentists or dental clinics).mp.

63. exp private practice/

64. exp family practice/

65. exp physicians/

66. exp physicians, family/

67. exp professional practice/

68. exp nurses/

69. exp nurse clinicians/

70. physician's practice patterns/

71. or/41-70

72. 20 and 40 and 71

73. limit 72 to english
