# Supplementary material for: Mechanisms and impact of public reporting on physicians and hospitals’ performance: A systematic review (2000–2020)
Source: PLoS One. 2021 Feb 24;16(2):e0247297. doi: 10.1371/journal.pone.0247297 (PMC7904172; doi:10.1371/journal.pone.0247297)
Supplement: S2 Appendix — (DOCX) [file pone.0247297.s003.docx]

**S2 Appendix**

**Screening guide**

| 1. **Empirical** |  |  |  |
| --- | --- | --- | --- |
| Is the paper an empirical study reporting primary data? | No | Yes | Doubt |
|  | Go to 5 | Go to 2 | Go to 2 |
| 1. **Quantitative data** |  |  |  |
| Does the paper report quantitative data? | No | Yes | Doubt |
|  | Go to 5 | Go to 3 | Go to3 |
| 1. **Study Design** |  |  |  |
| Is the study design a randomised controlled trial, quasi randomised trial, interrupted time series study, before and after study? Or a cohort, case-control, and cross-sectional study? | No | Yes | Doubt |
|  | Go to 5 | Go to 4a | Go to 4a |
| 1. **Association between public performance reporting and behavioural change** |  |  |  |
| a. Does the paper report exposure to public performance reporting? Including:   - Process measures - Healthcare outcomes - Structure measures - Consumer experiences - Expert and/or peer assessed measures | No | Yes | Doubt |
|  | Go to 5 | Go to 4b | Go to 4b |
| b. Does the paper report one or more of the following outcome measures?  Selection   - Changes in the healthcare decisions of purchasers - Changes in the healthcare decisions of providers - Changes in the healthcare decisions of consumers   Quality improvement   - Stimulated quality improvement activities   Clinical outcomes   - Resulted in improved clinical outcomes   Organisational change   - Resulted in changes to organisational structures and processes   Unintended consequences.   - Improving performance by treating patients who are less sick - Shifting resources to areas reported on - Gaming or manipulation of the data | No | Yes | Doubt |
|  | Go to 5 | Go to 4c | Go to 4c |
| c. Does the paper report an association between exposure to public performance reporting and behavioural change? Including either unadjusted association (e.g., correlations, unadjusted Odds Ratio, means and SDs, etc.) or adjusted associations (e.g., adjusted for one or more covariates, as well as models where public performance reporting is a mediator or a covariate) | No | Yes | Doubt |
|  | Go to 5 | Go to 5 | Go to 5 |
| 1. **Decision** |  | | |
| If all 1–4 ‘Yes’ | Include | | |
| If any 1–4 ‘No’ | Exclude | | |
| If any 1–4 ‘Doubt’ | Undecided – a third author to provide judgement | | |
