# Supplementary material for: Mechanisms and impact of public reporting on physicians and hospitals’ performance: A systematic review (2000–2020)
Source: PLoS One. 2021 Feb 24;16(2):e0247297. doi: 10.1371/journal.pone.0247297 (PMC7904172; doi:10.1371/journal.pone.0247297)
Supplement: S3 Appendix — (DOCX) [file pone.0247297.s004.docx]

**S3 Appendix**

**Risk of bias assessment**

**Cohort (n=8) and quasi-experimental studies (n=26)**

| **Authors and year** | **Representativeness of the exposed cohort** | **Selection of the non-exposed cohort*** | **Ascertainment of exposure** | **Demonstration that outcome of interest was not present at start of study** | **Comparability of cohorts on the basis of the design or analysis** | **Assessment of outcome** | **Was follow-up long enough for outcomes to occur** | **Adequacy of follow up of cohorts*** | **Stars rating** | **Overall strength of evidence** |
| --- | --- | --- | --- | --- | --- | --- | --- | --- | --- | --- |
| **Cohort studies** | | | | | | | | | | |
| Caron et al. 2004 | Truly representative of the average in the community | NA | Secure record | Yes | Study control for most important factor | Record linkage | Yes | Complete follow up | 7 | Moderate |
| Clough et al. 2002 | Truly representative of the average in the community | Drawn from a different source | Secure record | Yes | Study control for most important and additional factors | Record linkage | Yes | Subjects lost to follow up unlikely to introduce bias | 8 | Moderate |
| Daneman et al. 2012 | Truly representative of the average in the community | Drawn from the same community as the exposed cohort | Secure record | No | Study control for most important and additional factors | Record linkage | Yes | Subjects lost to follow up unlikely to introduce bias | 8 | Moderate |
| Mukamel et al.  2002 | Somewhat representative of the average in the community | NA | Secure record | No | Study control for most important and additional factors | Record linkage | Yes | Subjects lost to follow up unlikely to introduce bias | 7 | Moderate |
| Reineck et al. 2015 | Somewhat representative of the average in the community | Drawn from a different source | Secure record | Yes | Study control for most important and additional factors | Record linkage | Yes | Complete follow up | 8 | Moderate |
| Selvaratnam et al. 2020 | Truly representative of the average in the community | Drawn from the same community as the exposed cohort | Secure record | Yes | Study control for most important factor | Record linkage | Yes | Complete follow up | 8 | Moderate |
| Ukawa et al. 2014 | Selected group of users | Drawn from the same community as the exposed cohort | Secure record | Yes | Study control for most important and additional factor | Record linkage | Yes | Complete follow up | 8 | Moderate |
| Werner et al. 2008 | Truly representative of the average in the community | Drawn from the same community as the exposed cohort | Secure record | No | Study control for most important factor | Record linkage | Yes | Complete follow up | 7 | Moderate |
| **Quasi-experimental studies (before and after with/without control group)** | | | | | | | | | | |
| Dahlke et al. 2014 | Somewhat representative of the average in the community | Drawn from the same community as the exposed cohort | Secure record | Yes | Study control for most important and additional factors | Record linkage | Yes | Subjects lost to follow up unlikely to introduce bias | 9 | High |
| DeVore et al. 2016 | Truly representative of the average in the community | Drawn from the same community as the exposed cohort | Secure record | Yes | Study control for most important and additional factors | Record linkage | Yes | Complete follow up | 9 | High |
| Epstein 2010 | Truly representative of the average in the community | Drawn from a different source | Secure record | Yes | Study control for most important factor and additional factors | Record linkage | Yes | Complete follow up | 8 | Moderate |
| Hollenbeak et al. 2008 | Somewhat representative of the average in the community | Drawn from a different source | Secure record | Yes | Study control for most important and additional factors | Record linkage | Yes | Subjects lost to follow up unlikely to introduce bias | 8 | Moderate |
| Ikkersheim & Koolman 2012 | Truly representative of the average in the community | Drawn from the same community as the exposed cohort | Secure record | Yes | Study control for most important factor | Self-report | Yes | Complete follow up | 7 | Moderate |
| Joynt et al. 2016 | Truly representative of the average in the community | Drawn from the same community as the exposed cohort | Secure record | Yes | Study control for most important and additional factors | Record linkage | Yes | Subjects lost to follow up unlikely to introduce bias | 9 | High |
| Kraska et al. 2016 | Somewhat representative of the average in the community | Drawn from the same community as the exposed cohort | Secure record | Yes | Study control for most important factor | Record linkage | Yes | Complete follow up | 8 | Moderate |
| Leerapan 2011 | Selected group of users | Drawn from the same community as the exposed cohort | Secure record | No | Study control for most important and additional factors | Record linkage | Yes | Subjects lost to follow up unlikely to introduce bias | 7 | Moderate |
| Lind & Flug 2019 | Truly representative of the average in the community | Drawn from the same community as the exposed cohort | Secure record | Yes | Study control for most important and additional factors | Record linkage | Yes | Complete follow up | 9 | High |
| Mann et al. 2016 | Truly representative of the average in the community | NA | Secure record | Yes | Study control for most important and additional factors | Record linkage | Yes | Complete follow up | 8 | Moderate |
| Marsteller et al. 2014 | Somewhat representative of the average in the community | Drawn from the same community as the exposed cohort | Secure record | Yes | Study control for most important and additional factors | Record linkage | Yes | Subjects lost to follow up unlikely to introduce bias | 9 | High |
| Martin 2019 | Truly representative of the average in the community | Drawn from the same community as the exposed cohort | Secure record | Yes | Study control for most important and additional factors | Record linkage | Yes | No statement | 8 | Moderate |
| Mukamel et al.  2004 | Truly representative of the average in the community | Drawn from the same community as the exposed cohort | Secure record | Yes | Study control for most important and additional factors | Record linkage | Yes | Complete follow up | 9 | High |
| Vallance et al. 2018 | Truly representative of the average in the community | Drawn from the same community as the exposed cohort | Secure record | Yes | Study control for most important and additional factors | Record linkage | Yes | Complete follow up | 9 | High |
| Werner et al. 2010 | Truly representative of the average in the community | NA | Secure record | Yes | Study control for most important factor | Record linkage | Yes | Complete follow up | 7 | Moderate |
| Yamana et al. 2018 | Truly representative of the average in the community | Drawn from the same community as the exposed cohort | Secure record | Yes | Study control for most important and additional factors | Record linkage | Yes | Complete follow up | 9 | High |
| Yu et al. 2018 | Truly representative of the average in the community | NA | Secure record | No | Study control for most important and additional factors | Record linkage | Yes | Complete follow up | 7 | Moderate |
| **Quasi-experimental study (interrupted time series with/without control group)** | | | | | | | | | | |
| Baker et al. 2002 | Truly representative of the average in the community | NA | Secure record | Yes | Study control for most important and additional factors | Record linkage | Yes | No statement | 7 | Moderate |
| Baker et al. 2003 | Somewhat representative of the average in the community | NA | Secure record | Yes | Study control for most important factor | Record linkage | Yes | No statement | 6 | Moderate |
| Besley et al. 2009 | Truly representative of the average in the community | Drawn from a different source | Secure record | No | Study control for most important factor | Record linkage | Yes | Complete follow up | 6 | Moderate |
| Jang et al. 2011 | Truly representative of the average in the community | NA | Secure record | Yes | Study control for most important and additional factors | Record linkage | Yes | Complete follow up | 8 | Moderate |
| Noga et al. 2011 | Truly representative of the average in the community | NA | Written self-report | Yes | Study control for most important factor | Record linkage | Yes | Complete follow up | 6 | Moderate |
| Renzi et al. 2012 | Truly representative of the average in the community | Drawn from a different source | Secure record | Yes | Study control for most important and additional factors | Record linkage | Yes | Subjects lost to follow up unlikely to introduce bias | 8 | Moderate |
| Ryan et al. 2012 | Truly representative of the average in the community | Drawn from a different source | Secure record | Yes | Study control for most important and additional factors | Record linkage | Yes | Complete follow up | 8 | Moderate |
| Wang et al. 2014 | Truly representative of the average in the community | Drawn from the same community as the exposed cohort | Secure record | No | Study control for most important and additional factors | Record linkage | No | Complete follow up | 7 | Moderate |
| Werner et al. 2005 | Truly representative of the average in the community | Drawn from a different source | Secure record | Yes | Study control for most important and additional factors | Record linkage | Yes | Complete follow up | 8 | Moderate |

*Not applicable for study design without control group

**Cross-sectional studies (n=2)**

| **Authors and year** | **Representativeness of the sample** | **Sample size** | **Non-respondents** | **Ascertainment of the exposure** | **The subjects in different outcome groups are comparable, based on the study design or analysis. Confounding factors are controlled** | **Assessment of the outcome** | **Statistical test** | **Stars rating** | **Overall strength of evidence** |
| --- | --- | --- | --- | --- | --- | --- | --- | --- | --- |
| Bishop et al. 2012 | Truly representative of the average in the target population | Justified and satisfactory | Comparability between respondents and non-respondents characteristics is established, and the response rate is satisfactory | Validated measurement tool | The study control for most important and additional factors | Self-report | The statistical test used to analyse the data is clearly described and appropriate | 8 | Moderate |
| Smith et al. 2012 | Selected group of users | Justified and satisfactory | Comparability between respondents and non-respondents characteristics is established, and the response rate is satisfactory | Non-validated measurement tool but the tool is available or described | The study control for most important factor | Self-report | The statistical test used to analyse the data is clearly described and appropriate | 5 | Moderate |

**Randomised controlled trials (n=9)**

| **Authors and year** | **Random sequence generation** | **Allocation concealment** | **Blinding of participants and personnel** | **Blinding of outcome assessment** | **Incomplete outcome data** | **Selective reporting** | **Other bias** | **Summary assessment** | **Overall strength of evidence** |
| --- | --- | --- | --- | --- | --- | --- | --- | --- | --- |
| Fabbri et al. 2019 | Low risk | Low risk | Low risk | Low risk | Low risk | Low risk | Low risk | Low risk | High |
| Gourevitch et al. 2019 | Unclear risk | Unclear risk | Low risk | Low risk | High risk | Low risk | High risk | Unclear risk | Moderate |
| Ikkersheim & Koolman 2013 | Low risk | Unclear risk | Unclear risk | Unclear risk | Low risk | Low risk | High risk | Unclear risk | Moderate |
| Lui et al. 2016 | Unclear risk | Unclear risk | Low risk | Low risk | Low risk | Low risk | Low risk | Low risk | Moderate |
| Martino et al. 2012 | Low risk | Low risk | Unclear risk | Unclear risk | Low risk | Low risk | Low risk | Low risk | Moderate |
| Tang et al. 2016 | Low risk | Unclear risk | Low risk | Low risk | Unclear risk | Low risk | Low risk | Unclear risk | Moderate |
| Tang et al. 2017 | Low risk | Unclear risk | Low risk | Low risk | Unclear risk | Low risk | Low risk | Low risk | Moderate |
| Tu et al. 2009 | Low risk | Low risk | High risk | Low risk | Low risk | Low risk | Low risk | Unclear risk | Moderate |
| Yang et al. 2014 | Low risk | Unclear risk | Unclear risk | Unclear risk | Low risk | Low risk | Unclear risk | Unclear risk | Moderate |
