# Supplementary material for: Mechanisms and impact of public reporting on physicians and hospitals’ performance: A systematic review (2000–2020)
Source: PLoS One. 2021 Feb 24;16(2):e0247297. doi: 10.1371/journal.pone.0247297 (PMC7904172; doi:10.1371/journal.pone.0247297)
Supplement: S4 Appendix — (DOCX) [file pone.0247297.s005.docx]

**S4 Appendix**

**Data extraction for studies considered to be of low methodological quality following risk of bias assessment**

| **Authors and year** | **Country** | **Study design** | **Type of PPR** | **Level of reporting** | **Findings** |
| --- | --- | --- | --- | --- | --- |
| **User and provider response (selection)** | | | | | |
| Mukamel et al. 2000 (1) | US (New York) | cross-sectional study | New York State Cardiac Surgery Reports (CABG report cards) | Physician | 1. Do managed care organizations consider reported quality (RAMR rate, high/low performance outlier designation, volume) to select included physicians? (No effect, 80% of MCOs had no bias for higher/lower quality surgeons despite 90% stating that quality was their first/second most important consideration) |
| Pope 2009 (2) | US (14 states)^[[1]](#footnote-1)^ | cross-sectional study | US News and World Report hospital rankings | Hospital | 1. Increase in speciality non-emergency patient volume associated with 1-spot national USNWR specialty ranking improvement (Effect, positive, 0.88% p<0.01) 2. Increase in specialty non-emergency patient volume associated with 1-spot in-state USNWR specialty ranking improvement (Effect, positive, 6.8% p<0.01) |
| Maxwell and Temin 2002 (3) | US (National) | cross-sectional study | Health insurance carriers | Health plans | 1. Major companies are not using the managed competition approach (including quality information to make appropriate choice) to health care purchasing. Instead, most of the companies surveyed are purchasing health care in the same way as they do other inputs to production (industrial purchasing – minimise the cost of health care). |
| Emmert and Schlesinger 2017 (4) | US (National) | cross-sectional study | Any hospital report cards that respondents were aware of | Hospital | 1. Usage of report cards to search (Effect, 42% of all respondents, 55.6% of respondents aware of report cards) 2. Impact of report cards on hospital choice/influenced by report card to search for a hospital (Effect, 33.3% of all respondents and 80.3% of report card users) |
| **User and provider response (organisational quality improvement)** | | | | | |
| Pai et al. 2002 (5) | US (Michigan) | quasi-experimental study (before-after study) | Michigan Peer Review Organization (MPRO) profiling report | Hospital | 1. EF Documentation (Effect, positive, 5.4 percentage points p<0.05) 2. ACEI usage at discharge (No effect) 3. ACEI usage during stay or at discharge (Effect, positive, 2.2 percentage points p<0.05) |
| Hibbard et al. 2003 (6) | US (Wisconsin) | quasi-experimental study (before-after study) | QualityCounts report card | Hospital | 1. Quality improvement efforts in reported area of obstetrics (public-report group vs. private-report/no-report group, effect, positive, F=4.4, df=1.00, p<0.05) 2. Quality improvement efforts in reported area of cardiac (public-report group vs. private-report/no-report group, effect, positive, F=4.3, df=2.99, p<0.05) |
| Lamb et al. 2013 (7) | US (Wisconsin) | quasi-experimental study (before-after study) | Wisconsin Collaborative for Healthcare Quality website | Hospital | 1. Improvement In Aggregate Performance On Ambulatory Care Measures relative to first year reported (Effect, positive, range: 1.2-17.3 percentage points depending on measure) 2. Increase in HbA1c and LDL testing in patients with diabetes compared to control groups (Effect, positive, OR 1.05-1.07, p<0.05) |
| Emmert et al. 2016 (8) | Germany (not specified, likely national) | cross-sectional study | Jameda (patient review website) | Physician | 1. Implemented measures to improve patient care due to online ratings (Effect, 54.66% of respondents) |
| **Impact (clinical outcomes)** | | | | | |
| Hibbard et al. 2005 (9) | US (Wisconsin) | quasi-experimental study (before-after study) | QualityCounts report card | Hospital | 1. Percentage of hospitals with statistically significant improvements and declines in obstetric performance in the post-report period (Effect, positive, 1/3^rd^ of hospitals publicly reporting significantly improved performance relative to 1/4^th^ in private report group and ~13% in nonreporting group. 5% of publicly reporting hospitals declined in performance relative to 14% of private report group and 13% of nonreporting group 2. Improvement in performance for hospitals with worse than expected obstetric performance scores at baseline (Effect, positive, 7/8^th^’s of public reporting hospitals improved compared to ~1/3^rd^ of private report and nonreporting groups) 3. Improvement in performance scores relative to no report group (Effect, positive, t=2.25, df=92, p=0.3) |
| Snowden et al. 2012 (10) | US (Minnesota) | quasi-experimental study (before-after study) | Minnesota Health Care Disparities Report website | Medical groups | 1. Effect of public performance reporting on gap between outcomes for private/Medicare-insured patients and public MHCP patients (Effect, positive, gap narrowed for 5 out of 7 measures but only 1 statistically significant p<0.05 (childhood immunisation status); gap widened for 2 measures but only 1 statistically significant p<0.05 |
| **Impact (patient experience)** | | | | | |
| Elliott et al. 2010 (11) | US (all) | quasi-experimental study (before-after study) | Hospital Compare website (CMS Centres for Medicare & Medicaid Services) | Hospital | 1. Percentage of positive responses to 9 HCAHPS patient survey measures (Effect, 8 measures, 0.3-0.9 percentage points, p<0.001; No effect, doctor communication measure) |
| **Impact (clinical outcomes and patient experience)** | | | | | |
| Wang et al. 2018 (12) | US (National) | cross-sectional study | US News and World Report hospital rankings | Hospital | 1. 30-day RAMR for top-ranked vs non-ranked hospitals (Effect, positive, AMI, HF and CABG, 1-2.4 percentage points, p<0.001) 2. 30-day readmission for top-ranked vs non-ranked hospitals (No effect, AMI and CABG; Effect, negative, HF, 1.8 percentage points, p<0.001) 3. Patient satisfaction for top-ranked vs non-ranked hospitals (Effect, positive, 3.9 vs 3.3 out of 4, p<0.001) |
| **Both user and provider response (selection) and impact (clinical outcomes)** | | | | | |
| Jha and Epstein 2006 (13) | US (New York) | cohort study (retrospective) | New York State Cardiac Surgery Reporting System (CSRS) | Physician and hospital | 1. Ratings predicted subsequent RAMR for hospital (Effect, positive, RAMR = 1.59 top decile hospital vs 2.78 bottom decile) 2. Ratings predicted subsequent RAMR for physicians (Effect, positive, RAMR = 1.58 top decile physician vs. 3.20 bottom decile) 3. Hospital market share associated with performance (No effect) 4. Physicians with poor performance discontinuing CABG surgery practice (Effect, positive, within 2 years, 5% of bottom-quartile surgeons stopped practicing CABG surgery in NY state vs 5% of top 3 quartiles) |

ACEI angiotensin-converting enzyme inhibitors, AMI acute myocardial infarction, CABG coronary artery bypass grafting, EF ejection fraction, HbA1c haemoglobin, HF heart failure, LDL low-density lipoprotein cholesterol, MCO managed care organisation, MHCP Minnesota Health Care Programs, RAMR risk adjusted mortality rate, USNWR US News and World Report hospital rankings

**References**

1. Mukamel DB, Mushlin AI, Weimer D, Zwanziger J, Parker T, Indridason I. Do quality report cards play a role in HMOs' contracting practices? Evidence from New York State. Health Services Research. 2000;35(1 Pt 2):319.

2. Pope DG. Reacting to rankings: evidence from “America's Best Hospitals”. Journal of Health Economics. 2009;28(6):1154-65.

3. Maxwell J, Temin P, Law. Managed competition versus industrial purchasing of health care among the fortune 500. Journal of Health Politics, Policy. 2002;27(1):5-30.

4. Emmert M, Schlesinger M. Patients’ awareness, usage and impact of hospital report cards in the US. The Patient-Patient-Centered Outcomes Research. 2017;10(6):729-38.

5. Pai C-W, Finnegan GK, Satwicz MJ. The combined effect of public profiling and quality improvement efforts on heart failure management. The Joint Commission Journal on Quality Improvement. 2002;28(11):614-24.

6. Hibbard JH, Stockard J, Tusler M. Does publicizing hospital performance stimulate quality improvement efforts? Health Affairs. 2003;22(2):84-94.

7. Lamb GC, Smith MA, Weeks WB, Queram C. Publicly reported quality-of-care measures influenced Wisconsin physician groups to improve performance. Health Affairs. 2013;32(3):536-43.

8. Emmert M, Meszmer N, Sander U. Do health care providers use online patient ratings to improve the quality of care? Results from an online-based cross-sectional study. Journal of Medical Internet Research. 2016;18(9):e254.

9. Hibbard JH, Stockard J, Tusler M. Hospital performance reports: impact on quality, market share, and reputation. Health Affairs. 2005;24(4):1150-60.

10. Snowden AM, Kunerth V, Carlson AM, McRae JA, Vetta E. Addressing health care disparities using public reporting. American Journal of Medical Quality. 2012;27(4):275-81.

11. Elliott MN, Lehrman WG, Goldstein EH, Giordano LA, Beckett MK, Cohea CW, et al. Hospital survey shows improvements in patient experience. Health Affairs. 2010;29(11):2061-7.

12. Wang DE, Wadhera RK, Bhatt DL. Association of rankings with cardiovascular outcomes at top-ranked hospitals vs nonranked hospitals in the United States. JAMA Cardiology. 2018;3(12):1222-5.

13. Jha AK, Epstein AM. The predictive accuracy of the New York State coronary artery bypass surgery report-card system. Health Affairs. 2006;25(3):844-55.

1. Arizona, California, Colorado, Connecticut, Florida, Illinois, Iowa, Maryland, Massachusetts, New York, Pennsylvania, Virginia, Washington, Wisconsin [↑](#footnote-ref-1)
