# Supplementary material for: Mechanisms and impact of public reporting on physicians and hospitals’ performance: A systematic review (2000–2020)
Source: PLoS One. 2021 Feb 24;16(2):e0247297. doi: 10.1371/journal.pone.0247297 (PMC7904172; doi:10.1371/journal.pone.0247297)
Supplement: S5 Appendix — (DOCX) [file pone.0247297.s006.docx]

**S5 Appendix**

**Quality indicators reported in the studies**

| **Authors** | **Outcome indicators** | **Process indicators** | **Patient reported indicators** | **Other indicators** |
| --- | --- | --- | --- | --- |
| Mukamel et al. 2002 | Mortality (RAMR) |  |  | Number of patients treated |
| Mukamel et al. 2004 | Mortality (RAMR) | Number of CABG Procedures |  |  |
| Werner et al. 2005 |  | CABG surgery conducted, use of cardiac catheterization, use of PTCA |  |  |
| Epstein 2010 | Mortality (RAMR) |  |  |  |
| Martino et al. 2012 | Clinical Quality Scores (*Healthcare Effectiveness Data and Information Scores*) |  | Member Satisfaction (*Consumer Assessment of Health Care Providers and Systems tool*) |  |
| Ikkersheim & Koolman 2013 | Percentage of breast cancer operations where radical surgery is achieved during first surgery in breast saving therapy, percentage of patients who have a complication following eye surgery, percentage of decubitus ulcers with patients undergoing hip replacements, percentage deep wound infection hip/knee replacement (*Dutch Performance Indicators* for breath cancer, cataract surgery and hip and knee replacement) | Volume of patient undergoing surgery, percentage of patients who have a 28 days interval between cataract surgeries (*Dutch Performance Indicators* for breath cancer, cataract surgery and hip and knee replacement ) | Communication with surgeon/ ophthalmologist, communication with nurse, communication about medication, recommendation of hospital to family and friends *(Dutch Patient experience Indicators CQI tool)* | Structure: Number of surgeons in hospital who conduct breast cancer surgery |
| Yu et al. 2018 |  |  |  | Proportion of patients who received surgery at an excellent-performance hospital |
| Gourevitch et al. 2019 |  | Selection of hospital that met the Healthy People 2020 caesarean delivery rate |  |  |
| Fabbri et al. 2019 |  | Proportion of women who had 4+ antenatal care visits (i.e., attended a clinic or were visited at home by a healthcare worker) during their last pregnancy |  |  |
| Werner et al. 2008 |  | AMI (Aspirin at admission/discharge, ACE inhibitor for left ventricular dysfunction, beta-blocker at admission/discharge) HF (Assessment of left ventricular function, ACE inhibitor for left ventricular dysfunction), Pneumonia ( Oxygenation Assessment, Pneumococcal vaccination, timing of initial antibiotic therapy) |  |  |
| Besley et al. 2009 |  | hospital waiting times for elective hospital admissions |  |  |
| Bishop et al. 2012 |  | Preventive (smoking cessation counselling, BMI screening, weight reduction counselling, urinalysis), diabetes (blood pressure measurement), heart failure ( 2 prescriptions made), Coronary Artery Disease (2 prescriptions), general (no antibiotic prescriptions, prescription for atrial fibrillation, prescription for COPD) |  |  |
| Leerapan 2011 | Optimal Diabetes Care scores depending on 1) haemoglobin A1c level, 2) blood pressure, 3) LDL-cholesterol level, 4) daily aspirin use and 5) documented tobacco-free status |  |  |  |
| Jang et al. 2011 |  | Caesarean section rates |  |  |
| Renzi et al. 2012 |  | Adjusted proportions for PCI, hip fractures operated within 48 hours & caesarean deliveries |  |  |
| Smith et al. 2012 |  | Diabetes improvement interventions n=22 (9 directed at patient: Patient phone/mail/e-mail reminders for diabetes appointments/tests, Formal one-on-one patient education, Formal group patient education classes, Support groups for diabetic patients; 3 directed at provider: Provider reminders for diabetes care, provider education on diabetes, Provided diabetes care quality data to providers; 10 directed at the system: Adopted guidelines for diabetes care, Registry or list of diabetes patients, Adopted specific set of pre-determined diabetes orders, Procedures for alternate prescribers, Routine diabetes progress communications, Specialty physician added to the care team, Multidisciplinary care teams, Group visits, Care manager for diabetic patients, Patients see multiple care providers in one visit) |  |  |
| Wang et al. 2014 |  | Injection prescribing rates |  |  |
| Yang et al. 2014 |  | Antibiotics prescription rates for upper respiratory tract infections |  |  |
| Ukawa et al. 2014 |  | 5 AMI process measures (aspirin at admission/discharge, Beta-blockers at admission/discharge, ACEI or ARB use) |  | Hospital characteristics as co-variates: ownership, case volume, number of cardiovascular specialists per AMI patient, disclosure status, teaching hospital) |
| Kraska et al. 2016 | Achieving recanalization target in percutaneous coronary intervention (PCI) with the indication “acute coronary syndrome with ST elevation up to 24 h” | n=5 process indicators for pacemaker implantation, gynaecological surgery, and obstetrics (guideline-compliant indication for bradycardia, guideline-compliant system selection for bradycardia, antibiotic prophylaxis in hysterectomy, presence of paediatrician at premature births, antenatal corticosteroid therapy in premature births with prepartum hospitalization for at least two calendar days) |  |  |
| Liu et al. 2016 |  | Antibiotic prescription rate, Injection prescription rate, Average patient prescription expenditure |  |  |
| Tang et al. 2016 |  | Prescriptions containing antibiotics, Prescriptions containing two or more antibiotics, Prescriptions containing injections, Prescriptions containing antibiotic injections, Average prescription cost |  |  |
| Tang et al. 2017 |  | Prescriptions requiring antibiotics, Prescriptions requiring 2+ antibiotics, Prescriptions requiring injection antibiotics |  |  |
| Lind & Flug 2019 |  | Proportion of Medicare beneficiaries receiving conservative therapy before MRI of the lumbar spine |  |  |
| Baker et al. 2002 | Risk adjusted in-hospital mortality, early post-discharge mortality, 30-day mortality for AMI, CHF, gastrointestinal haemorrhage, COPD, pneumonia and stroke |  |  |  |
| Clough et al. 2002 | General hospital mortality (RAMR), intensive care unit mortality |  |  |  |
| Baker et al. 2003 | 30-day mortality (RAMR) for patients with or AMI, HF, gastrointestinal haemorrhage, obstructive pulmonary disease, pneumonia and stroke |  |  |  |
| Caron et al. 2004 | Mortality for AMI, CHF and stroke | Length of stay for AMI, CHF and stroke. Primary caesarean delivery rates, total caesarean delivery rates, vaginal birth after caesarean delivery rates |  |  |
| Hollenbeak et al. 2008 | In-hospital mortality (RAMR) for AMI, CHF, haemorrhagic stroke, ischemic stroke, sepsis |  |  |  |
| Noga et al. 2011 | Falls and falls with injury rates in acute care hospitals |  |  |  |
| Ryan et al. 2012 | 30-day mortality for heart attack, heart failure and pneumonia |  |  |  |
| Daneman et al. 2012 | Clostridium difficile Infection Rates |  |  |  |
| Marsteller et al. 2014 | Central line associated bloodstream infections (CLABS |  |  |  |
| Devore et al. 2016 | 30-day readmission and 30-day post-discharge mortality for MI, HF, pneumonia, COPD and diabetes |  |  |  |
| Joynt et al. 2016 | 30-day mortality (RAMR) for 15 common nonsurgical discharge diagnoses (e.g. AMI, HF, pneumonia) |  |  |  |
| Martin 2019 | Mortality for HF and AMI | Length of stay for HF and AMI |  |  |
| Ikkersheim & Koolman 2012 |  |  | Physician communication, nurses communication, medication communication, treatment, pain treatment, treatment explanation, feeling safe, respect for autonomy, contradictive information, discharge information, ward intake experience, intake conversation, hospital accessibility, accommodation, nurse service, intervention planning (Dutch CQI Patient Experience Indicators) |  |
| Mann et al. 2016 |  |  | Satisfaction with physician communication |  |
| Tu et al. 2009 | 30-day and 1-year mortality for AMI, STEMI, Non-STEMI, and CHF | n=12 AMI process indicators (use of standard admission orders, left ventricular function assessment, lipid test on arrival, fibrinolytic or PCI, fibrinolytic administration decided by emergency department physician, fibrinolytic given prior to transfer to CCU or ICU, Aspirin on arrival/discharge, beta-blockers on arrival/discharge, ACE inhibitors or ARB for left ventricular dysfunction, statin at discharge), n=6 CHF process indicators (left ventricular function assessment, daily weights recorded, counselling CHF, ACE inhibitor or ARB for left ventricular dysfunction, beta-blockers for left ventricular dysfunction, Warfarin for atrial fibrillation)(*Feedback for Effective Cardiac Treatment Tool)* |  |  |
| Werner et al. 2010 | 30-day mortality, 30-day readmission (both risks adjusted) for ACI, HF and Pneumonia | Length of stay, AMI (Aspirin at admission/discharge, ACE inhibitor for left ventricular dysfunction, beta-blocker at admission/discharge) HF (Assessment of left ventricular function, ACE inhibitor for left ventricular dysfunction), Pneumonia (Oxygenation Assessment, Pneumococcal vaccination, timing of initial antibiotic therapy) |  |  |
| Reineck et al. 2015 | Post-acute care use, acute care hospital transfer rates, in-hospital and 30-day mortality rates | Discharge pattern |  | Structure: hospital characteristics (no of beds in hospital/ICU, Teaching status, Ownership, Metropolitan Statistical Area size) |
| Yamana et al. 2018 | In-hospital mortality for AMI | Aspirin within 2 days of admission |  |  |
| Selvaratnam et al. 2020 | Perinatal mortality (stillbirth and neonatal mortality) | Rate of detection of severely small-for-gestational-age (SGA) single babies |  |  |
| Dahlke et al. 2014 | n=16 (mortality and readmission for Heart attack, heart failure and pneumonia, mortality or serious complication after elderly surgery, Colorectal surgery or lower extremity bypass surgery | n=32 (11 heart attack or chest pain measures, 4 heart failure measures, 6 pneumonia care measures, 11 surgical care improvement process measures) | n=10 Patient experience measures (*HCAHPS tool*) hospital tracked and communicated clinical health information, hospitals disseminated reports to the community on quality and costs of service | Structure: n=20 hospital characteristics (hospital ownership/control, number of beds, number of admissions and surgical operations, number of operating rooms, commission on Cancer accreditation, Joint Commission accreditation, Membership in the Council of Teaching Hospitals) |
| Vallance et al. 2018 | 90-day RAMR for elective major colorectal resections | Surgeon risk-averse behaviour (patient predicted 90-day mortality rate) |  |  |

ACE Angiotensin Converting Enzyme; ACEI Angiotensin Converting Enzyme Inhibitors; ARB angiotensin-receptor blockers; AMI Acute Myocardial Infarction; BMI Body Mass Index; CABG Coronary Artery Bypass Graft; CHF Congestive Heart Failure; COPD Chronic Obstructive Pulmonary Disease; HCAHPS Hospital Consumer Assessment of Healthcare Providers and Systems; HF heart failure; ICU Intensive Care Unit; PCI Percutaneous Coronary Intervention; PTCA Percutaneous Transluminal Coronary Angioplasty; STEMI ST-Elevation Myocardial Infarction; RAMR Risk Adjusted Mortality Rate
