# Supplementary material for: Mechanisms and impact of public reporting on physicians and hospitals’ performance: A systematic review (2000–2020)
Source: PLoS One. 2021 Feb 24;16(2):e0247297. doi: 10.1371/journal.pone.0247297 (PMC7904172; doi:10.1371/journal.pone.0247297)
Supplement: Attachment — Submitted filename: Response to reviewers.docx [file pone.0247297.s007.docx]

**Reviewer #1: Recent paper by Prang et al. named “Mechanisms and impact of public reporting on physicians and hospitals' performance: A systematic review (2000-2016)” tried to summarize the impact of PPR on physicians and hospitals’ performance. The overall idea behind this research is legitimate with strong worldwide interest in the topic but the general impression is that the manuscript could be improved by better specifying some crucial elements. Paper itself is without a strong conclusion, introducing the topic with the statement “However, previous studies have demonstrated inconsistent effects of PPR, potentially due to the various PPR characteristics examined.” and concluding in the same manner: “There was limited and inconclusive evidence to demonstrate a relationship between PPR and patient experience.” This should be modified.**

*Thank you for your comment. Based on an updated search of the literature, we have now included an additional 15 studies, resulting in a total of 45 studies in the systematic review. We have amended our conclusion in the discussion to reflect this update and made a clearer statement about the impacts of PPR. The findings suggested that there were moderate positive effects of PPR for both selection and quality improvement. Similarly, there were moderate positive impact of PPR on clinical outcomes and patient experience (albeit the low number of three studies for patient experience).*

**Overall, the introduction needs to better explain the problematic behind the need to have this kind of research especially in respect to the complexity of the topic, should be discussed furthermore with stronger arguments for conducting this kind of research. Further, there any several already available (and much more structured) reviews published and authors should emphasize why conducting other, especially since conclusions remained more-less the same as in previously published reviews on the topic. Also, considering the study design here, it could easily be an umbrella-review? Could authors explain why they didn’t opt for this kind of review since again there are already several reviews available which they considered for the additional reference search?**

*Whilst several reviews have been undertaken to identify research relating to PPR, the magnitude and type of impacts of PPR were rarely considered. This is because previous reviews focused on summarising different aspects of PPR (mechanisms or impacts) without differentiating between the various healthcare settings/contexts. The impacts of PPR are highly dependent of what the objectives and who the targeted audiences are (see table below), and these are likely to vary across healthcare settings and conditions. As such, differentiating between the mechanisms and impacts of PPR by audience types for specific contexts/conditions will provide greater conceptual clarity surrounding the impacts and utility of PPR. This is a strong justification for a further systematic review. Based on an update of the literature search, we were able to distinguish the impacts of PPR by conditions/procedures (coronary artery bypass graft and percutaneous coronary intervention), health plans (selection/switching), and physicians/hospitals performance (selection and quality improvement), which is the focus of this review. This rationale has now been included on pages 4 and 5 of the introduction with the table below.*

*Classification of public performance reporting by mechanisms and audiences*

| **Quality improvement (performance)** | **Organisation or practitioner subject to PPR** | **Performance measures subject to PPR** |
| --- | --- | --- |
|  | Hospitals (units in hospitals)  Medical specialists  Health plans (e.g. HMOs)  Family physicians (general practitioners) | Clinical indicators  Structure indicators  Process indicators  Treatment indicators  Patient outcomes (e.g. mortality)  Patient experience |
| **Selection (services)** | **Consumer, organisation, or practitioner responding to PPR** | **Choice of services** |
|  | Consumers  Hospitals (units in hospitals)  Medical specialists  Health plans (e.g. HMOs)  Family physicians (general practitioners) | Hospitals (units in hospitals)  Medical specialists  Health plans (e.g. HMOs)  Family physicians (general practitioners) |

**In the introduction author mention two pathways through with PPR can improve quality of care, when there are actually three pathways suggested in the literature, third is the reputation pathway introduced by Hibbard et al (2005). This should be modified.**

*We have described the third pathway on page 3 of the introduction: “In the reputation pathway, PPR can negatively affect the public image of a provider or an organisation. Reputational concerns will therefore motivate providers or organisations to protect or improve their public image by engaging in quality improvement activities”.*

**Methods: Why there was a time-limit year 2000? Also, search should be updated after 16th April 2015 until 2020 since several studies have been published and this might give different conclusions. Or at least reasons for not doing so should be documented. Inclusion and exclusion criteria are well defined and PRISMA guide was followed.**

*We limited our search from 2000 and onwards because the practice of PPR before then was significantly different. This is because of the widespread use of PPR online in the 2000s. This has now been noted on page 6 of the methods.*

*As suggested by the reviewer, we have updated the search to include additional PPR studies published from 2016 to 2020. We identified a total of 9,603 articles across five databases. Following titles and abstracts screening, we excluded 9,572 articles, leaving 31 articles for full-text screening. Following full-text screening and risk of bias assessment, 15 additional articles were included. This resulted in a total of 45 articles in the present systematic review.*

**Results section: should be synthesised a bit more, following the common pattern and not just retelling findings from the tables.**

*We presented the findings by user groups (consumers and providers) and PPR mechanisms (selection and quality improvement), and impact of PPR (clinical outcomes and patient experience). Given the heterogeneity of the studies, we attempted to describe the effects across the various conditions/contexts as concisely as possible, as well as providing additional relevant information not reported in Table 2.*

**Discussion: Most of the finding have already been described in the previous reviews, with perhaps difference of those reported previously that here some healthcare settings and types of providers were excluded (!?). Finally, the main question that remains unanswered – what should one do with these findings? This should be clearly summarized - what this research adds to the previous already available? Also, policy implications and/or further recommendations are lacking here, these should be added.**

*Thank you for your comment. We have now included a policy implications section on pages 24 and 25 of the discussion. Identifying who the intended audiences and what the objectives of PPR are key to increasing the effectiveness of PPR. We described the prerequisites conditions for consumers and healthcare providers that health policy makers should considered when implementing PPR. Understanding the healthcare system in which PPR operates in and identifying system-level and socio-cultural barriers will help increase the uptake and impact of PPR.*
