# Supplementary material for: Mechanisms and impact of public reporting on physicians and hospitals’ performance: A systematic review (2000–2020)
Source: PLoS One. 2021 Feb 24;16(2):e0247297. doi: 10.1371/journal.pone.0247297 (PMC7904172; doi:10.1371/journal.pone.0247297)
Supplement: Attachment — Submitted filename: responses to reviewers comments.docx [file pone.0247297.s008.docx]

**Please address all issues raised by the reviewer, with a special focus on the exclusion of studies because of the low quality. This is a crucial point: in general, no study should be excluded because of an evaluation based on a biased tool such as the Newcastle Ottawa scale. Thus, all studies should be discussed, and their limitations mentioned and considered. Also, the report of the findings of the individual studies is too scarce, and quantitative estimates of effects should be reported. This will also help understanding whether it was really impossible to perform a meta-analysis, of it might have been performed on some specific outcomes.**

*Although some risk of bias can be drawn from the methodological quality score, the Cochrane Collaboration^[1]^ perceives a summary quality score to be a subjective judgement and may be potentially misleading as it ascribed equal weight to each of the nominated criteria. Given there is a lack of consensus on which is the best procedure or tool to assess the methodological quality of observational studies, the NOS was considered to be appropriate despite assigning a total score to each study. We acknowledged that the methodological quality of the studies should be interpreted with caution. This has been included on page 24 of the limitation section of the manuscript.*

*As per reviewer’s comment #2, we have included an additional supplementary table of the studies we considered to be of low methodological quality following a risk of bias assessment. We have also included the direction of the effects with the corresponding estimates in Table 2.*

**Reviewer #1: Compliments to the authors for addressing previously raised issues and for these additional efforts when updating the literature search which all together significantly improved quality of the research. I’m very much pleased to see that the number of included studies increased 50% which further reflects the importance of this topic, where in just 4 years for which the search was now extended the authors found 15 new eligible studies.**

1. **Here are some minor, mostly technical, modifications that should be amended before final decision:
   Introduction was enriched with several good explanations bringing it to more… also, adding table 1 with classification of PR by mechanisms and audience helps reader having a better picture of the topic. Even though during this update of the literature search you have probably included all the available eligible studies covered by the following reviews (published in years after 2016), but for the sake of comprehensiveness these should also be acknowledged (after double checking it) in the search diagram (Fig.2) in the part "additional records identified through previous reviews". You have already cited most of these reviews throughout the discussion so it's unclear why this is not presented also in the diagram.**

*Thank you for your comment. We have merged Figures 1 and 2 together.*

2. **Several studies with assigned low quality have been excluded in the step “Full-text articles assessed for eligibility” which is not in line with the exclusion criteria reported in the methods. Usually, evaluation of the methodological quality of studies comes after the final selection of the eligible studies since here it’s very easy to slip into biased conclusion and some potentially valuable conclusions, based on quality data, might come even from studies with low(er) methodological quality. Finally, I would encourage authors to keep these studies, perhaps in a separate supplementary table so the readers could have a general (and full) idea of the available evidence, while not considering findings from these low quality studies when making the main conclusion here. In case the authors decide to go with the current setting, the low quality of studies should be clearly stated as the exclusion criteria.**

*Thank you for your comment. The risk of bias assessment was conducted following the full-text assessment. This has now been clarified on page 6 of the manuscript. The exclusion of low methodological quality studies has been included in the exclusion criteria. The data extraction for the excluded studies considered to be of low methodological quality following a risk of bias assessment have been included as a supplementary table.*

3. **I wasn’t able to find the supplementary table with QA of the included studies using Newcastle-Ottawa Scale nor the Cochrane Collaboration’s tool for assessing risk of bias? If not placed somewhere where I might’ve missed it, this should be added in the supplementary tables. Also, in the text please always refer to the “methodological” quality in sentences when describing quality of the studies.**

*The risk of bias table has been included as a supplementary table. Sentences where ‘quality’ referred to the quality of the studies, have been replaced with ‘methodological quality’ throughout the manuscript.*

4. **Please consider placing Figure 1 and Figure 2 into one, the reader doesn’t need to be bothered with two diagrams since this is an issue of this study design of this study (search of the literature repeated several times).**

*As suggested, we have merged Figure 1 and 2 together.*

5. **Description of the studies – lines 187/188, please try rephrasing when summing up the studies “by two from Canada and Japan…. by one from India, Germany…” instead if listing one by one, in general would be better to synthesize this descriptive part for the reader.**

*The sentence has been amended to: “Studies were predominantly conducted in the US (n=26), followed by five from China, two from Canada, Japan, the Netherlands, and the UK, one from Australia, Germany, India, Italy, Korea, and Taiwan”.*

6. **Table 2 could benefit from some restyling. First, please insert references next to study. Also, try perhaps collapsing some cells to avoid repetition (for mechanism, etc.) or perhaps divide table into segments by mechanism (since you seem to order studies by this) or similar to have it more compact, save space and make it easier to read. Also, it would be good to have the direction of the effect (positive, negative, etc.) in the findings column. It might be useful to state the city/province/state if available for a country where the study was conducted, since healthcare system might be organized differently if country is decentralized, like in case of Italy or USA, Swiss, etc.**

*Thank you for your comments. Table 2 has been amended:*

- *The mechanism/impact and mechanism/impact type columns have been deleted. Headers denoting the mechanism and impact type for each section have been included instead.*
- *The direction of the effects has been included in the ‘findings’ column, with the corresponding estimates and where possible with the 95% CI and p-values.*
- *The references for each study have been added.*
- *The city/province/state for each study has been added to the ‘country’ column.*

7. **Results - try rephrasing sentences where you number the studies, i.e. line 224 and later: “7 of 7 studies… 1 of 2 studies…” across the text, since this seems copy-pasted, monotonous for reading, try sum it up a bit. Perhaps try all studies (if 2 of 2), 50% of studies, majority of studies, etc. to make it more diverse and dynamic for readers. I highly appreciate that you divided results in segments by mechanism, much clearer now and easier to navigate.**

*Thank you for your suggestions. We have amended the sentences to:*

- *“In summary, half of the studies reported positive effects of PPR, 1 reported a negative effect and the rest no effect. These findings suggest moderate level of evidence for PPR and selection of patients, physicians and hospitals.”*
- *“In summary, all studies reported positive effects of PPR for primary care (although the findings of three studies appeared to be derived from one RCT), and half of the studies reported positive effects of PPR for outpatients and for hospitals. These findings suggest strong and moderate level of evidence for PPR and quality improvement activities in primary care and hospitals, respectively but inconclusive evidence for outpatients given the low number of studies.”*
- *“In summary, the majority of the studies reported positive effects of PPR on clinical outcomes including mortality (7 of 15) and readmission rates, infection rates and falls (4 of 6), and patient experience (2 of 3). These findings suggest moderate level of evidence for PPR and clinical outcomes and some evidence for PPR and patient experience, albeit the low number of studies.”*

8. **Discussion – in line 279 was stated “Unlike previous reviews [13, 15, 19, 22], we found that PPR positively influenced consumers’ (i.e. patients, providers, purchasers) selection of healthcare providers (i.e. individual physician, hospital)..” while previously in results line 183 you wrote that 50% demonstrated positive effect: “In summary, 5 of 10 studies reported positive effects of PPR, with 1 of 10 a negative effect and 4 of 10 no effect. These findings suggest moderate level of evidence for PPR and selection of patients, physicians and hospitals.” This should be modified in the light of your findings. Also, some of the previous reviews did find positive effect (with smaller number of included studies in respect to here) on patients’ choice of surgeons, of healthcare plan, of nursing home, etc.**

*Previous reviews have reported positive effects of PPR on selections of health plans (Fung et al. 2008; Chen et al. 2010; Vukovic et al. 2017). However, the effects of PPR on selection of hospitals and individual providers remain unclear with some reviews reporting positive effects (Chen et al. 2010; Vukovic et al. 2017), and others mixed or no effects (Fung et al. 2008; Metcalfe et al. 2018). The difference may be due to the inclusion of nursing homes, and hospital volume and market share as proxy of selection. We chose to report the findings of health plans in a separate review and excluded nursing homes from the review because the choices consumers are asked to make are vastly different and the characteristics of PPR including what indicators are publicly reported are likely to vary substantially.*

*This has been clarified and the paragraph has been amended to: “The majority of studies showed that PPR positively influenced the selection of healthcare providers (i.e. individual physician, hospital) by patients, providers, and purchasers. This is consistent with the findings of reviews conducted by Chen et al. [15] and Vukovic et al. [19] but not others [13, 22]. The discrepancy between reviews likely reflects the healthcare choices consumers and healthcare providers are asked to make, as some reviews incorporated selection of healthcare providers, health plans and nursing homes together, and used hospital’s surgical volume and market share as measures of selection. All studies included in our review focused on actual consumer choice behaviour in the hospital and physician sector of health services. The findings related to the selection of health plans [20] and market share associated with CABG/PCI [21] are reported separately.*

9. **Discussion needs references insertion across text, i.e. lines 301-316 are completely lacking in references even though several hypothesis and examples are mentioned, these should be appropriately inserted.**

*References have been included.*

10. **Implications segment – I’m very pleased to read this part, it is very useful and significantly increased the quality and applicability of the research.**

*Thank you.*

11. **Funding - please state if the funder (Medibank Better Health Foundation) had some role in any of the steps during this research (design of the study, selection & data collection, interpretation, etc.).**

*The research was funded by Medibank Better Health Foundation. Views expressed are those of the authors and not the funding agency. The funding agency had not role in study design, data collection and analysis, decision to publish, or preparation of the manuscript. This has now been included on page 26 of the manuscript in the funding section.*

1. Higgins JP, Altman DG. Assessing risk of bias in included studies. In: Higgins JP, Green S, editors. Cochrane handbook for systematic reviews of interventions. London: Wiley; 2008.
