# Supplementary material for: Mechanisms and impact of public reporting on physicians and hospitals’ performance: A systematic review (2000–2020)
Source: PLoS One. 2021 Feb 24;16(2):e0247297. doi: 10.1371/journal.pone.0247297 (PMC7904172; doi:10.1371/journal.pone.0247297)
Supplement: Attachment — Submitted filename: response to reviewers.docx [file pone.0247297.s009.docx]

**Reviewer #1: Thank you for considering previous suggestions and for modifying the manuscript.
Here are some minor changes to be conducted before the final decision is made:**

1. **Not essential, but since last time it was also underlined by the Editor „quantitative estimates of effects should be reported. This will also help understanding whether it was really impossible to perform a meta-analysis, of it might have been performed on some specific outcomes.“ I feel it would be more appreciated to have quantitative estimates of the effect in a separate column (just numeric values) so the reader doesn’t have to search across the text in the column “Findings”, and so it would be much easier to understand and compare the effect among the studies, and also the possibility of conducting a meta-analysis.**

Thank you for your suggestion. The quantitative estimates have been included in a separate column.

2. **In lines 140-141 Authors say: „The methodological quality of each study was graded as very low, low, moderate or high (see S3 Appendix).“ But the numerical value for the cut off points to classify the Overall strength of evidence is not reported (i.e. how many stars were considered for the classification of high, moderate, etc.)?**

For cohort and quasi-experimental studies (maximum of 9 stars can be awarded), we considered 9 stars as high quality, 6-8 stars as moderate quality, and less than 5 stars as low quality. For cross-sectional study design (maximum of 10 stars can be awarded), we considered 9-10 stars as high quality, 5-8 stars as moderate quality and less than 4 stars as low quality. This has been reported on page 7 of the manuscript.

3. **Let’s just say you did not considered studies with low quality in the synthesis (so we don’t create confusion) but still have extracted the data rather than excluding (ignoring) them, thus the sentence „Studies considered to be of low methodological quality were excluded from the synthesis (see S4 Appendix)“ should be modified somehow for reader to know that these information is still available in the Supplementary file (now when you wrote “see S4 Appendix” it seems like just the list of these studies is available) since usually when you say they are excluded you don’t extract data, so better to underline this. I also feel this sentence belong to the Results section now, since data was extracted (perhaps in lines 170-171?)**

The sentence has been clarified to denote that data extraction for the low-quality studies are available in S4 appendix. The sentence has been added in the data extraction and synthesis section on page 7 of the manuscript.

4. **Make sure that dissertations included (line 187) were peer-reviewed before making it available to the public since this might introduce bias?**

Although the published PhD dissertations may have not been peer-reviewed, they would have been thoroughly examined by two examiners as part of the completion requirements for the award of a PhD degree prior to being made publicly available. All the PhD dissertations were completed at an American University.

5. **Table 2 further suggestions:**

**• Please modify the title of the Table2 appropriately (i.e. characteristics, main findings or so...)**

The title of Table 2 has been changed to “characteristics and main findings of included studies”.

**• Column „Country“ should be named also region/state/city or so...**

This has been amended.

**• Type of PPR - try to define few main types of PPR to make it more uniform and easier to follow – in general, there are few main types of PPR (report cards, survey results, or so) and more details could be added in the parenthesis, i.e. Report Cards (Cleveland Health Quality Choice). Now it seems there are so many different types, which is not true. Also some types are not clear which type they are (i.e. Patients First (voluntary public reporting))?**

Thank you for your suggestion. Types of PPR include: report cards; reports; surveys; bulletin boards and brochures; posters; and website.

**• CABG acronym needs explanation below table**

CABG acronym has been included.

**• Perhaps consider grouping columns „lever of reporting „ and „type of PPR“ into one column, since there are only few information on „level of reporting“ instead of repeating the information. Or some symbol (asterisk or so) can just be added for physician/hospital/clinic to save space and make table more appealing and clear.**

Thank you for your suggestion. Superscripts have been included in “the type of PPR” column to denote the level of reporting where ^c^ indicates hospital, ^d^ physician, and ^e^ clinic.

**• Level of reporting „village“ for Fabbri et al. is unclear? It refers to what? Since then in lines 169-170 you wrote: “Fabbri et al. [47] reported no effects of PPR on the selection of hospitals..”**

In Fabbri et al.’s study, the report cards contain information on village-level indicators of maternal and neonatal healthcare in Uttar Pradesh, India. The primary outcome was the proportion of women who had at least four antenatal care visits.

In the sentence “Both Gouveritch et al. [46] and Fabbri et al. [47] reported no effects of PPR on the selection of hospitals with lower caesarean delivery rates by pregnant women and maternal and neonatal health care services, respectively”, selection of hospitals referred to Gouveritch et al.’s study, whilst maternal and neonatal healthcare services referred to Fabbri et al.’s study. The sentence has been amended for clarification: “Gouveritch et al. [46] reported no effects of PPR on the selection of hospitals with lower caesarean delivery rates by pregnant women. Similarly, Fabbri et al. [47] reported no effects of PPR on the proportion of women who received maternal and neonatal health care services.*”*

**• In column „Findings“ when you report „no effect“ - it means there was no statistically significant effect or there was no difference at all after the PPR? In case of non stat. sign. findings, the measures of effect should still be reported (even if not significant). This would still allow the possibility of conducting a meta-analysis in case of at least two studies with common outcome and effects reported.**

No effect refers to no statistically significant effect of PPR. This has been clarified in Table 2. Data extraction for studies who reported no effect of PPR have been conducted and included in Table 2.

**• When reporting the effect, it should be uniformly presented in a consistent way (effect, 95% CI, p-value) it’s rather strange to not have them all reported in the original studies (i.e. OR should always be accompanied by 95% CI and p-value), please also check suppl. files of the original papers. This should be modified across the column „Findings“. Decimals should be always reported in the same way (rounded to two or three numbers after the comma)**

Studies reported various estimates including odds ratio, incidence rate ratio, percentage and percentage points. Where possible, we attempted to report the estimates (to 2 decimal points), SE, 95% CI and exact p-value. Unfortunately, not all studies reported SE, 95% CI or p-value and some studies reported estimates up to 1 decimal point only.

**• Also, as mentioned above, quantitative estimates of the effect would appeal better in a separate column.**

A separate column has been added for quantitative estimates.

6. **Further comments:
   • Line 225, these three studies (ref. 60, 61, 62) are “conducted on the same population” (this clarification should be added in some way next to the RCT to make it clear).**

The references of the three studies have been added.

**• Across the text, numbers less than 10 are usually written in words**

This has been amended throughout the manuscript.

**• Since in the Acknowledgment you only mentioned Dr. Angela Zhang “for conducting risk of bias assessment and data extraction of studies from the third search” I was just wondering is the process of methodological quality evaluation conducted by just dr. Angela Zhang or was it conducted by two reviewers after which their evaluations were confronted, as you stated in lines 128-132 for screening titles and abstracts? Excluding studies based on the level of assessed methodological quality is a critical point of this review, and it must be conducted as objectively as possible in order not to introduce bias into your conclusions.**

Khic-Houy Prang and Hana Sabanovic conducted the third search. Both reviewed titles, abstracts and full-text articles. If discrepancies occurred between them, David Dunt made the final decision. The risk of bias and data extraction of each of the full-text articles were then conducted by two researchers (Khic-Houy Prang, David Dunt or Angela Zhang). The methods section and acknowledgment section have been clarified.
